# Hypothalamic obesity in pediatric vs. adult craniopharyngioma: mechanisms, management, and cardiometabolic outcomes

**DOI:** 10.1007/s11102-026-01760-z

**Published:** 2026-09-27

**Authors:** Khushal Gupta, Maryam Zeinali, David Altschul, Vijay Agarwal, Michael Karsy

**Affiliations:** 1 The Leo M. Davidoff Department of Neurological Surgery, Albert Einstein College of Medicine, Montefiore Einstein, 111 East 210th Street, New York, NY 10467 USA; 2 Mazandaran University of Medical Sciences, Sari, Iran; 3 Department of Neurosurgery, University of Michigan, Ann Arbor, MI USA

**Keywords:** Hypothalamic obesity, Craniopharyngioma, Pediatric, Neuroinflammation, GLP-1 receptor agonists, Cardiometabolic outcomes

## Abstract

**Purpose:**

Hypothalamic obesity (HO) is a treatment-related complication in craniopharyngioma (CP) survivors that markedly impairs long-term health and quality of life. Although overall survival exceeds 90% for both pediatric and adult CP, HO prevalence and clinical course differ substantially by age at onset. We aimed to compare HO manifestations, mechanisms, and treatment response between pediatric and adult CP cohorts.

**Methods:**

We conducted a scoping review of studies published between 2000 and 2025, comparing pediatric (childhood-onset) versus adult-onset CP cohorts, classified by age at diagnosis. Data on obesity prevalence, mechanistic pathways, therapeutic interventions, and cardiometabolic outcomes were extracted and synthesized.

**Results:**

In pediatric CP survivors, obesity rates rise from 5.7% pre-treatment to 57% post-treatment; in adults, from 19.2% to 29.2%. HO severity correlates with the extent of hypothalamic damage by Puget and QST grading. Key mechanisms include disrupted melanocortin signaling, central leptin and insulin resistance, and neuroinflammation via resistin–TLR4 and NLRP3 pathways. GLP-1 receptor agonists (exenatide, semaglutide) have produced marked weight loss in small case series (up to 17% over 6 months with semaglutide), but randomized trials did not meet their primary weight outcomes and direct pediatric-versus-adult comparisons are lacking. Bariatric procedures yield moderate success; sleeve gastrectomy achieves 5-year weight loss comparable to that in non-CP controls (21.7% vs. 21.8%), whereas Roux-en-Y gastric bypass is less effective than in controls (22.7% vs. 32.0%). Both cohorts face significant long-term risks: 2.2-fold higher all-cause mortality and 9.3-fold higher diabetes risk in adults, non-alcoholic fatty liver disease in 47% of pediatric survivors, metabolic dysfunction-associated fatty liver disease in 67.4% of adults, and insulin resistance in approximately 30% of pediatric patients.

**Conclusion:**

HO following CP shows distinct age-dependent trajectories in prevalence, underlying mechanisms, and clinical course; suggested age-related differences in treatment response remain to be confirmed in direct comparative studies. Tailored management integrating mechanistic insights and age-specific therapies is critical to mitigating the cardiometabolic burden in these patients.

## Introduction

Craniopharyngiomas (CP) are rare sellar pathologies with low histological malignancy but significant potential for neurovascular injury affecting both pediatric and adult populations. Despite high overall survival rates of approximately 92%, quality of life is frequently compromised by hypothalamic obesity (HO), a devastating complication resulting from hypothalamic injury [1]. Obesity prevalence markedly increases from 5.7% pre-diagnosis to 57% postoperatively in some studies of pediatric CP, highlighting the profound metabolic dysfunction that can occur with treatment [2]. Tumor origin, growth pattern, and surgical approach significantly influence HO development, with weight gain typically accelerating during the first postoperative year [3, 4]. 

Notably, the pathophysiological mechanisms and clinical presentation of HO differ between pediatric and adult populations, necessitating age-specific approaches to management (Table 1). HO is characterized by disruption of multiple regulatory pathways controlling energy intake, expenditure, autonomic dysfunction, and hormonal signaling. Recent advances in pharmacological approaches (GLP-1 receptor agonists, central stimulants, metformin), bariatric interventions, and specialized lifestyle modifications show promise but demonstrate variable efficacy between age groups [5–7]. 

**Table 1:** Prevalence and Weight Trajectory in Craniopharyngioma Patients

Characteristic	Pediatric Population	Adult Population
Pre-diagnosis obesity prevalence	5.7% [2]	19.2% [3]
Post-treatment obesity prevalence	57–60% [2, 101]	29.2% [3]
Weight gain trajectory	Rapid increase during first postoperative year [3, 12]	40.8% of patients experience ≥ 5% weight gain post-surgery [3]
Average weight gain	Progressive weight gain (median BMI increase + 4.59 SD with hypothalamic involvement vs. +1.20 SD without) [1]	Mean increase of 2.7 kg (*p* = 0.043) [44]
Risk factors for weight gain	Hypothalamic involvement, posterior hypothalamic damage [12]	Lower preoperative BMI [OR 0.78, 95% CI (0.67–0.90)], adamantinomatous subtype [OR 3.46, 95% CI (1.02–11.76)] [3]
Body composition	Lower fat-free mass (62.4% vs. 67.5%) and higher fat mass (37.5% vs. 32.5%) compared to multifactorial obesity [41]	Limited specific data on body composition in adults

This scoping review uniquely compares HO manifestations between pediatric and adult CP patients, addressing an important gap in the current literature. Long-term cardiometabolic impact, morbidity and mortality in CP survivors are discussed [8]. By comparing pediatric versus adult treatment paradigms and studies, we aim to provide evidence-based guidance for age-appropriate management strategies while identifying critical directions for future research to improve quality of life in this challenging disease.

## Methods

This review was conducted as a scoping review, with the aim of mapping the breadth of the evidence on HO in pediatric and adult CP populations rather than formally appraising the quality of individual studies. PubMed/MEDLINE was searched for literature published between January 2000 and May 2025, using combinations of disease descriptors (“craniopharyngioma”, “hypothalamic obesity”, “hypothalamic injury”) with terms for population (“pediatric”, “childhood-onset”, “adult-onset”), mechanisms (“leptin”, “melanocortin”, “neuroinflammation”), management (“GLP-1 receptor agonist”, “setmelanotide”, “bariatric surgery”, “lifestyle intervention”), and outcomes (“obesity”, “metabolic syndrome”, “cardiovascular”, “quality of life”). Searches were conducted iteratively during the drafting of the review rather than under a single prospectively registered protocol, and were supplemented by hand-searching of the reference lists of included articles and of recent reviews; a formal count of records screened is therefore not reported.

Eligible sources were English-language publications reporting on the epidemiology, pathophysiology, management, or cardiometabolic and psychosocial outcomes of HO in patients with CP, including randomized trials, prospective and retrospective cohort studies, cross-sectional studies, case series and case reports, systematic and narrative reviews, and selected conference abstracts reporting emerging data. Preclinical and mechanistic studies were included where relevant to pathophysiology or investigational therapies. Records were excluded if they were not available in English or did not report outcomes relevant to HO in CP or related suprasellar lesions. Titles and abstracts were screened for relevance, followed by full-text review, and findings were charted narratively by topic (epidemiology, mechanisms, management, and long-term outcomes). In keeping with scoping-review methodology, no formal risk-of-bias assessment was performed.

Throughout this review, “pediatric” refers to childhood-onset CP (diagnosed before 18 years of age) and “adult” to adult-onset CP (diagnosed at 18 years or older), classified by age at CP diagnosis unless otherwise specified. Where cited cohorts comprised adults with childhood-onset disease, or transitional-age participants (15–25 years), this is stated explicitly. Because no prospective study has directly compared pediatric and adult cohorts, the age-group comparisons presented in this review are indirect, being drawn from separate studies that differ in populations, follow-up duration, tumor characteristics, and outcome definitions; they should therefore be interpreted with caution.

## Pathophysiological mechanisms of hypothalamic obesity

### Clinical neuroanatomical considerations

The extent of hypothalamic injury, quantified via Puget grading, independently predicts obesity risk across ages (Table 2). The Puget grading system defines tumor proximity to the hypothalamus: Grade 0 (no involvement of 3rd ventricle floor), Grade 1 (hypothalamic displacement anterior to mammillary bodies), and Grade 2 (hypothalamic disruption) [9, 10]. Pediatric patients with surgical hypothalamic lesions exhibit 2.94-fold higher odds of obesity at last follow-up (95% CI 1.73–5.08), with posterior hypothalamic involvement conferring particular risk [11]. There remains an ongoing debate between gross total resection vs. subtotal resection with radiotherapy to preserve hypothalamic function [13]. Similar debates regarding pituitary stalk preservation aim to minimize patient morbidity as an important determinant of quality of life.

**Table 2:** Pathophysiological Differences of Hypothalamic Obesity

Mechanism	Pediatric Population	Adult Population	Common Features
Hypothalamic damage	More vulnerable due to developing neural circuits; surgical hypothalamic lesions carry 2.94-fold higher odds of obesity [11]	May have less neuroplasticity to compensate; structural damage predicts weight gain [3, 103]	Graded by Puget system (0–2); damage to hypothalamic nuclei disrupts energy homeostasis [9, 10, 32]
Eating behavior	Pronounced hyperphagia and impaired satiety; altered food attention and satiety signaling [2, 27]	Similar hyperphagia but potentially different hunger score responses to treatment (−2.3 with exenatide in CRANIOEXE trial) [59]	Disruption of melanocortin-4 receptor (MC4R) signaling pathways [63]
Metabolic adaptation	Dramatic metabolic shift during growth and puberty; parental BMI influences outcomes [31]	Distinct metabolic signatures with alterations in uric acid levels and specific lipid species [45]	Central insulin and leptin resistance; autonomic dysregulation [79]
Insulin dynamics	Hyperinsulinemia and insulin hypersecretion in response to OGTT [104]	More pronounced β-cell dysfunction	Autonomic dysregulation of β-cell function [104]

Surgical approach significantly influences weight outcomes, with open craniotomy associated with greater BMI increases compared to transsphenoidal approaches (*p* = 0.002), attributed to more invasive tumors or greater disruption of hypothalamic pathways [9]. Multiple systematic reviews and meta-analyses report superior outcomes for endoscopic endonasal approaches compared to transcranial surgery in craniopharyngioma management [14, 15]. In these observational series, the endoscopic approach was associated with higher gross total resection rates (89.8% vs. 77.3%, *p* < 0.05) and lower rates of hypothalamic dysfunction [16]. Specifically, endoscopic surgery is associated with reduced hypopituitarism (53.4% vs. 68.1%, *p* < 0.05) and diabetes insipidus (51.1% vs. 72.4%, *p* < 0.05) compared to transcranial approaches [16]. A meta-analysis of pediatric craniopharyngioma patients revealed that endoscopic endonasal approaches achieved higher gross total resection rates (OR = 5.25, 95% CI 1.21–22.74) and lower recurrence rates (OR = 0.54, 95% CI 0.31–0.94) with reduced hypopituitarism (OR = 0.34, 95% CI 0.12–0.97) [17]. These comparisons should nonetheless be interpreted with caution: they derive from observational cohorts and their meta-analyses in which the surgical approach was not randomly assigned. Tumors selected for endoscopic endonasal resection tend to be smaller, more midline, and less invasive, whereas larger tumors with lateral or ventricular extension are preferentially approached transcranially; selection bias, together with confounding by tumor size, location, hypothalamic involvement, and surgical expertise, may therefore account for part of the observed differences [14, 15]. Accordingly, the association between endoscopic approaches and lower rates of hypothalamic dysfunction and hypopituitarism should not be interpreted as establishing causality [14–17]. Postoperative hypothalamic damage assessment also uses a similar grading system, evaluating the involvement of third ventricle floor [10, 13]. 

Several additional anatomical classification frameworks have been proposed to optimize preoperative planning and predict hypothalamic morbidity. The QST classification system, for instance, categorizes tumors based on origin and spatial relation to the hypothalamic–pituitary axis, Type Q (pituitary region), Type S (pituitary stalk), and Type T (tuberal region/third ventricle floor), each with distinct implications for hypothalamic disruption and surgical complexity [18, 19]. Notably, Type T tumors show pia mater disruption and finger-like projections into the third ventricle floor, correlating with higher hypothalamic injury risk [20]. Another novel endoscopic classification based on intraoperative stalk relationship further divides tumors into Peripheral and Central types. Peripheral tumors (71.7%) show greater potential for stalk preservation, with subtypes such as hypothalamic stalk CPs being most prone to hypothalamic damage [19]. In contrast, Central tumors, aligned strictly along the stalk, have the lowest stalk preservation rate (15.4%) and highest hypothalamic risk [19]. Another system stratifies tumors by their topographic growth patterns, Group I (infrasellar), Group II (suprasellar extraventricular), and Group III (suprasellar subpial/ventricular) [21]. Group III tumors, involving both children and adults, are strongly associated with higher rates of postoperative weight gain and high-grade hypothalamic dysfunction, with 41.7% of children and 16.7% of adults showing significant weight increases [21]. Furthermore, classifications based on hypothalamic location (Type A: infra-hypothalamic; Type B: perforating; Type C: supra-hypothalamic) inform surgical route selection: endonasal approaches for Types A/B and transcranial for Type C [22]. Outcomes correlate with location: long-term disease control was achieved in 100% of Type A, 92% of Type B, and 83% of Type C cases, with papillary CP distribution highest in Type C [22]. 

### Age considerations

Neuroanatomical differences and age-related variations in hypothalamic plasticity may significantly influence obesity outcomes following CP treatment (Table 2). Studies report that preoperative obesity rates are notably higher in adolescents than in younger children (*p* < 0.05), suggesting developmental differences in hypothalamic vulnerability and energy regulation. Although the pediatric hypothalamus exhibits greater neuroplasticity, severe structural injury, particularly to key nuclei such as the ventromedial hypothalamus and arcuate nucleus, results in persistent dysregulation of satiety and metabolic control. This paradox highlights how extensive anatomical disruption can overwhelm intrinsic repair mechanisms, with long-term consequences for energy homeostasis and quality of life [23–25]. 

### Anatomical considerations

The severity of HO exhibits a strong anatomical correlation with the degree of baseline hypothalamic dysfunction. Patients with radiologically confirmed hypothalamic involvement demonstrate significantly higher BMI both at diagnosis (*p* = 0.001) and during follow-up (*p* < 0.001) compared to those without such involvement [26]. Moreover, functional baseline capacity is protective against postoperative BMI elevation in patients with hypothalamic lesions (*p* < 0.001), suggesting that the degree of hypothalamic disruption plays a role in physical and metabolic functioning. Recent neuroimaging and behavioral studies confirm that structural hypothalamic damage alters central processing of attention, satiety, and reward, thereby driving pathological eating behaviors and promoting weight gain [27]. Among CP patients, rates of HO reach up to 60%, with obesity severity directly linked to the grade of hypothalamic damage [28]. 

Severity of HO also correlates with the extent and location of hypothalamic damage [29]. Posterior hypothalamic involvement is significantly associated with more severe obesity outcomes [11, 30]. This finding is seen in both pediatric and adult patients. While pituitary hormone deficiencies commonly coexist with HO, there is endocrinological outcome heterogeneity depending on patient age and tumor characteristics. While most studies focus predominantly on pediatric populations, limited data suggest age-dependent differences in HO development. Age at diagnosis (< 6 years versus ≥ 6 years) influences event-free survival and quality of life [11]. Notably, baseline parental BMI also influences obesity development in pediatric craniopharyngioma survivors, though its impact is smaller than that of hypothalamic damage [31]. 

### Neuroendocrine and satiety signaling disturbances

**Fig. 1:**
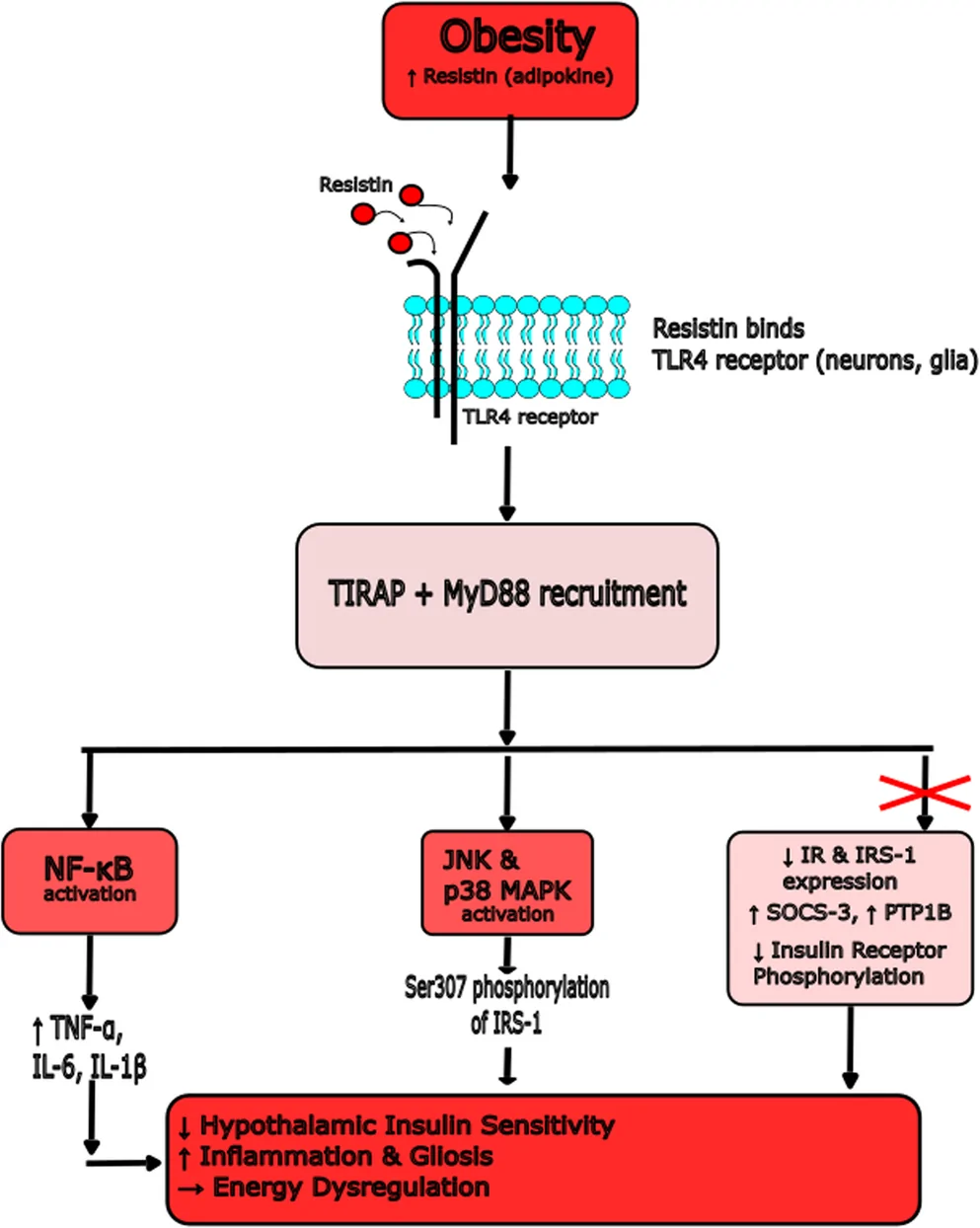
Resistin/Toll-like receptor 4 (TLR4) signaling and its role in hypothalamic insulin resistance. This pathway represents a critical link between obesity-induced inflammation and hypothalamic dysfunction. Obesity leads to elevated levels of resistin, an adipokine that binds to TLR4 receptors on hypothalamic neurons and glial cells. This triggers recruitment of the TIRAP–MyD88 complex and activates downstream inflammatory cascades, including NF-κB and JNK/p38 MAPK pathways. NF-κB activation promotes proinflammatory cytokines (TNF-α, IL-6, IL-1β), while JNK/p38 MAPK activation leads to inhibitory phosphorylation of IRS-1 and reduced insulin receptor signaling. These changes collectively reduce hypothalamic insulin sensitivity, promote gliosis, and disrupt energy homeostasis. IL: interleukin; MAPK: mitogen activated protein kinase; TIRAP: TIR domain containing adaptor protein; TNF: tumor necrosis factor; TLR: toll-like receptor

HO is characterized by persistent hyperphagia, where patients experience perpetual hunger without normal meal-induced satiety (Table 2) [32]. CP patients also show higher food inattention, lower satiety, and altered eating behaviors compared to controls [27]. The pathophysiology involves an inability to regulate hormonal signals, effectively mimicking a state of central nervous system starvation [33]. Recent evidence suggests that inflammatory mediators in CP cyst fluid also contribute to this dysregulation [34]. CXCL1, an inflammatory mediator seen in cyst fluid, can disrupt normal satiety signaling pathways. These inflammatory mediators activate the NF-κB pathway and upregulate suppressor of cytokine signaling 3 (SOCS3) expression, leading to leptin resistance even without structural hypothalamic injury [34]. This finding challenges traditional understanding of HO mediated solely by hypothalamic damage, by demonstrating that chemical factors from the tumor itself can induce metabolic dysfunction through inflammatory pathways, creating leptin resistance that parallels the severity observed in patients with physical hypothalamic damage [34]. Recent insights also implicate innate immune signaling pathways in perpetuating hypothalamic inflammation and metabolic dysfunction. One such mechanism involves the resistin/Toll-like receptor 4 (TLR4) signaling pathway, which is upregulated in obesity. Resistin, an adipokine elevated in obese states, binds to TLR4 receptors on hypothalamic neurons and glial cells, activating NF-κB and MAPK pathways [35, 36]. These cascades promote inflammation, impair insulin receptor signaling, and contribute to hypothalamic insulin resistance and energy dysregulation (Fig. 1).

**Fig. 2:**
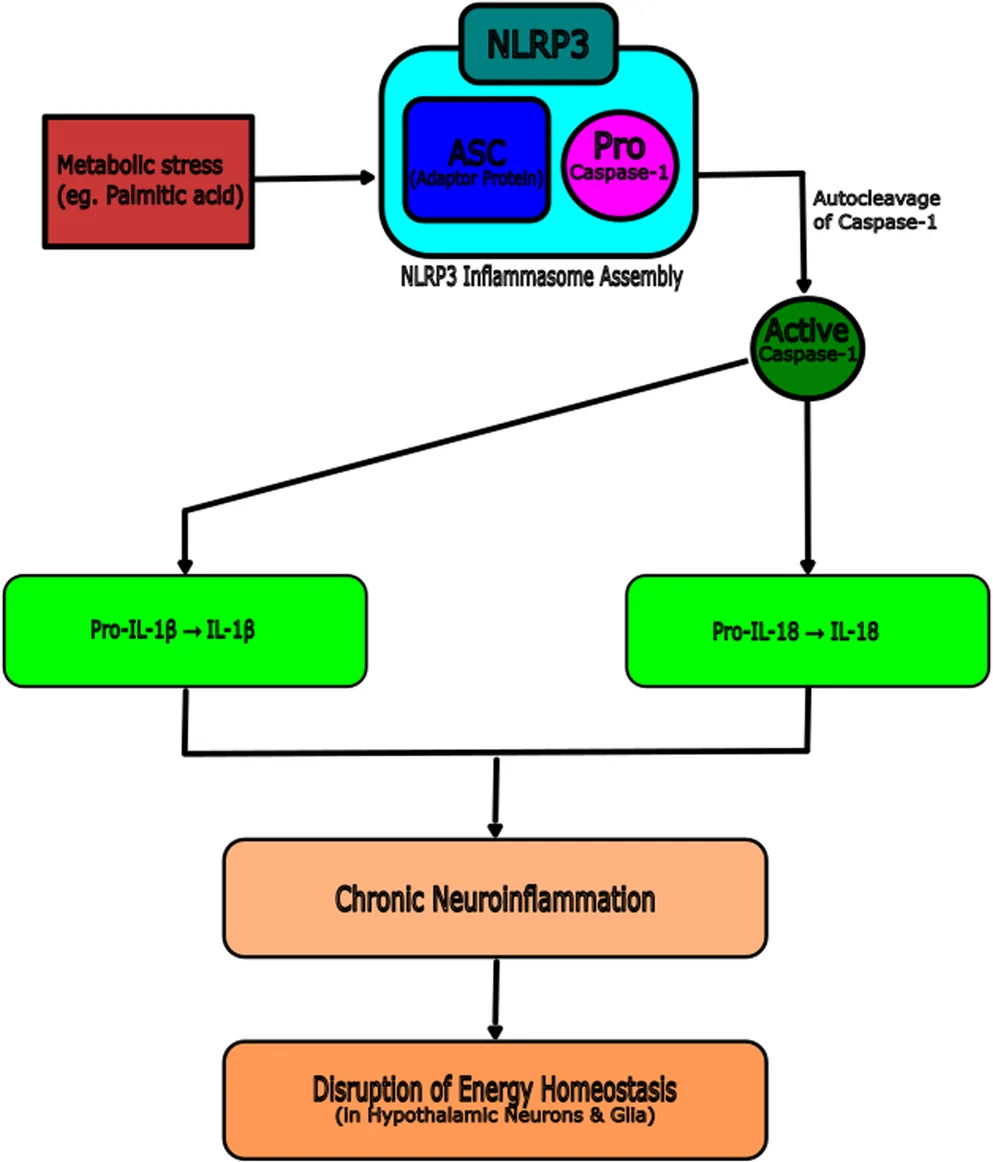
NLRP3 inflammasome activation and its role in chronic neuroinflammation and energy imbalance. The NLRP3 inflammasome is a key mediator of inflammation-driven metabolic dysfunction in hypothalamic obesity. Metabolic stressors such as saturated fatty acids (e.g., palmitic acid), which are elevated in obesity, trigger the assembly of the NLRP3 inflammasome complex in hypothalamic neurons and glial cells. This complex consists of NLRP3, the adaptor protein ASC, and pro-caspase-1. Upon activation, caspase-1 undergoes autocleavage and promotes the maturation and secretion of pro-inflammatory cytokines IL-1β and IL-18. IL: interleukin; NLRP3: NOD-like receptor pyrin domain-containing protein 3. Abbreviations: CP, craniopharyngioma; HO, hypothalamic obesity; QST, Q-pituitary/S-stalk/T-tuberal classification

Another critical but often underrecognized inflammatory mechanism is the NLRP3 inflammasome pathway, which links metabolic stress with neuroinflammation. Saturated fatty acids like palmitic acid activate the NLRP3 inflammasome complex (NLRP3, ASC, and caspase-1), resulting in cleavage and release of IL-1β and IL-18 [37–39]. These cytokines promote chronic inflammation within the hypothalamus, disrupting energy homeostasis even after initial tumor resection. This mechanism may help explain persistent inflammation in craniopharyngioma survivors despite stable anatomical outcomes (Fig. 2).

Adult CP patients exhibit significantly elevated fasting leptin levels (27.9 ± 34.2 vs. 11.9 ± 11.6 µg/l) compared to patients with non-functioning pituitary adenomas, even when corrected for fat mass percentage [40]. Adult CP patients show significantly lower fasting ghrelin levels (131 ± 129 vs. 191 ± 119 ng/l) compared to controls with non-functioning pituitary adenomas [40]. Both pediatric and adult populations with CP-related HO experience disruption of melanocortin signaling pathways, resulting in hyperphagia, central insulin and leptin resistance, decreased sympathetic activity, and low energy expenditure [32]. However, the developmental stage at which this disruption occurs significantly influences the resulting metabolic phenotype.

In summary, the pathophysiology of HO involves a multifaceted interplay of mechanisms. Central to its development is structural damage to hypothalamic nuclei, particularly posterior regions, which disrupt neural regulation of hunger, satiety, and energy balance. Superimposed on this are inflammatory processes, such as microglial activation, which may perpetuate neuronal dysfunction and impede hormonal signaling. Additionally, neuroendocrine circuit dysregulation, especially involving the leptin–melanocortin pathway, contributes to central leptin resistance and metabolic imbalance. While these mechanisms are shared across age groups, their relative impact may vary between pediatric and adult patients, influenced by developmental plasticity, hormonal status, and the timing of injury [29, 31]. 

### Age-specific metabolic alterations and tumor biology

Children with CP-induced HO exhibit significantly altered body composition that differs from typical childhood obesity, suggesting unique pathophysiological mechanisms (Table 2) [41]. Children with CP-related obesity are characterized by lower fat-free mass percentage (62.4% vs. 67.5%) and higher fat mass percentage (37.5% vs. 32.5%) [41]. This pattern differs from adult CP patients, who show a 40.8% rate of clinically meaningful weight gain (≥ 5%) within 12 months of surgery, with particular vulnerability among those with lower preoperative BMI [3]. 

Age at diagnosis significantly influences metabolic outcomes due to the importance of endocrinological contributions to growth and development. Iughetti et al. demonstrated that younger age at diagnosis is itself an independent risk factor for subsequent BMI increase (likely because the hypothalamus is still undergoing rapid growth and myelination in early childhood, making it more vulnerable to surgical and radiation-induced injury), and in the CCSS cohort, female brain tumor survivors diagnosed at ≤ 9 years had a three-fold higher risk of obesity than those diagnosed later [42]. De Alcubierre et al. highlight that craniopharyngiomas presenting during childhood and adolescence coincide with critical windows for pituitary and pubertal hormone maturation; deficiencies in GH, sex steroids, and other pituitary axes emerging or persisting through the transition age (15–25 years) directly exacerbate long-term metabolic derangements [43]. Sterkenburg et al. described a median diagnosis age of 8.1 years in their childhood-onset cohort and demonstrated that those with hypothalamic involvement had persistent, severe obesity (median BMI-SDS increase + 4.59 SD vs. + 1.20 SD in those without hypothalamic involvement), alongside enduring neuropsychological impairments into adulthood (e.g., lower physical-functioning and cognitive scores) [1]. 

In a consecutive series of 120 adult-onset craniopharyngioma patients, Wu et al. used multivariable logistic regression (adjusting for age at diagnosis, gender, follow-up time, preoperative BMI, radiotherapy and hypothalamic involvement) to show that the adamantinomatous subtype was independently associated with clinically meaningful weight gain (adjusted OR 3.46, 95% CI 1.02–11.76, *P* = 0.047, with papillary subtype as reference) [3]. This suggests that the more aggressive growth pattern, calcifications and cystic structure of adamantinomatous tumors often necessitate extensive hypothalamic dissection, exacerbating injury to appetite-regulating nuclei (e.g., arcuate and ventromedial) and driving greater postoperative weight gain.

Adult CP patients exhibit age-specific metabolic and biochemical signatures. These profiles include alterations in uric acid levels and lipid species such as diacylglycerol DG(18:2/20:4) [45], which have been associated with impaired insulin sensitivity and increased cardiometabolic risk. Clinically, such changes suggest that adult HO patients may follow a different metabolic trajectory compared to pediatric cases, with greater susceptibility to components of metabolic syndrome, including hyperlipidemia, insulin resistance, and NAFLD. In contrast, pediatric CP survivors demonstrate early, severe metabolic derangements: Srinivasan et al. (2004) showed significantly greater abdominal adiposity (*P* = 0.008), higher fasting triglycerides (*P* = 0.02), lower HDL-to-total-cholesterol ratios (*P* = 0.015), and insulin resistance versus matched controls [46]. Jung et al. (2017) reported a 46.8% prevalence of NAFLD in children with HO, with 27.3% of cases diagnosed within one year post-surgery [47]. Hoffmann et al. (2015) found a 50% prevalence of NAFLD in long-term childhood-onset CP survivors with hypothalamic involvement, with ~ 30% exhibiting severe steatosis and associated fatigue and metabolic syndrome features [48]. Dogra et al. (2022), in an adult-onset cohort (*n* = 91), observed postoperative weight gain of 9.5 ± 14.8 kg (*P* < 0.001), obesity rising from 40.5% to 62%, and impaired glucose metabolism from 17.4% to 34% over ~ 100 months [49]. Chen et al. (2025) studied 242 adult-onset CP patients, reporting a 67.4% prevalence of MAFLD and, among those with MAFLD, a 32.5% risk of advanced fibrosis (BMI OR 1.51 per 1 kg/m²; 95% CI 1.33–1.72; *P* < 0.001) [50]. These findings underscore the need for age-tailored metabolic monitoring and interventions, especially in adults, where standard pediatric-derived HO protocols may not fully address the evolving metabolic burden.

## Management strategies for hypothalamic obesity

### Conservative approaches

Dietary interventions remain a cornerstone in managing HO in CP patients (Table 3). Well-balanced restrictive caloric diets (30% fat, 45% carbohydrates, 25% protein) and various hypocaloric diets (8–10 kcal/cm/day) are both feasible in pediatric patients, although long-term efficacy is variable [51]. However, traditional dietary approaches often fail to produce meaningful and sustained body mass index reductions in HO patients despite being considered therapeutic interventions [5]. In pediatric populations, 92% of caregivers report dietary modifications, though only 31% find calorie restriction effective [2]. Adults exhibit marginally better adherence to hypocaloric regimens, only partially due to greater self-regulation capacity, yet long-term weight stabilization remains elusive [3, 52]. Self-monitoring technologies, such as food diaries, calorie-tracking apps, and fitness wearables, can support conservative management of HO by improving dietary awareness and accountability [53]. 

**Table 3:** Management Approaches and Their Efficacy

Treatment Approach	Efficacy in Pediatric Population	Efficacy in Adult Population	Notes
Dietary Interventions	92% of caregivers try dietary modifications; 31% find calorie restriction effective vs. 69% for carbohydrate modification [2]	Better adherence to hypocaloric regimens but limited long-term effectiveness [105]	Well-balanced restrictive diets (30% fat, 45% carbohydrates, 25% protein) and hypocaloric diets (8–10 kcal/cm/day) are most common approaches [105]
Physical Activity	Despite fatigue, daytime sleepiness, and impaired motor activation, 73% tried exercise (52% found it helpful) and 37% tried supervised programs (45% beneficial) [2]	Often limited by musculoskeletal comorbidities; low-impact activities preferred [53]	Self-monitoring technologies may improve adherence when implemented with clinical support [52]
GLP-1 Receptor Agonists	Exenatide reduces energy intake (−430 kcal) during buffet meals; promising results in youth (ages 10–26) [60, 61]	CRANIOEXE trial: weight reduction with exenatide (−3.8 kg) vs. placebo (−1.6 kg), though difference not statistically significant (*p* = 0.11) [59]	Recent case series of semaglutide in 4 females: 17% weight loss (20.2 kg) over 6 months with improved energy levels and mobility [58]
Other Pharmacological	Octreotide stabilizes weight (+ 1.6 kg vs. +9.1 kg with placebo); improves quality of life [104]	Limited specific adult data on non-GLP-1 agents	Diazoxide-metformin combination, [62] setmelanotide (MC4R agonist), [63, 72] and central stimulants such as dextroamphetamine [106] show promise, particularly in pediatric cases
Bariatric Surgery	Generally reserved until growth completion; requires specialized pediatric expertise [69, 74]	Five-year weight loss: 22.0% in craniopharyngioma patients vs. 29.5% in controls (*p* = 0.02) [71]	RYGB less effective in craniopharyngioma patients (22.7% weight loss) vs. controls (32.0%), but sleeve gastrectomy shows similar outcomes (21.7% vs. 21.8%) [71]

When hunger and satiety cues are impaired, self-monitoring tools are most effective when combined with ongoing clinical support, such as regular input from dietitians or behavioral therapists. Without such guidance, patients often struggle to maintain motivation and consistency, limiting the long-term success of self-monitoring strategies [54]. The effectiveness of dietary interventions in HO appears to be significantly enhanced when tailored to the individual’s specific pathophysiological profile. Given that HO arises from distinct neuroanatomical damage, particularly to nuclei involved in satiety and energy regulation, standard calorie-restriction approaches often yield suboptimal results. Interventions that account for impaired hypothalamic signaling, disrupted leptin–melanocortin pathways, and altered energy expenditure may better address the unique metabolic challenges in HO patients. Personalized strategies may include structured meal timing, low-glycemic index diets to reduce hyperinsulinemia, and behavioral reinforcement techniques, especially in combination with pharmacologic or supportive therapies [29]. 

Barriers to physical activity implementation differ markedly between age cohorts. Pediatric patients with HO often experience hypothalamic-mediated fatigue, excessive daytime sleepiness, and impaired motor activation, which limit spontaneous physical engagement [2, 55]. In one survey, 73% of children with CP-related HO had attempted exercise interventions, with 52% of those who tried exercise finding it helpful for weight management [2]. When examining supervised exercise programs specifically, 37% of children had trialed such interventions, with 45% of those participants reporting beneficial effects [2]. These findings suggest that while exercise interventions show promise in pediatric HO management, their effectiveness remains modest and may require individualized approaches that account for the underlying pathophysiological barriers characteristic of hypothalamic dysfunction [2, 55]. 

Adults frequently contend with musculoskeletal comorbidities which may limit high-intensity regimens, favoring low-impact activities [54]. In pediatric patients, behavioral interventions demonstrate strong family influence, with parental BMI correlating with offspring adiposity trajectories (maternal *r* = 0.28, paternal *r* = 0.30) [31], whereas adult outcomes rely more on individualized cognitive-behavioral strategies [29]. 

### Pharmacological management

#### GLP-1 receptor agonists: mechanisms and efficacy evidence

Glucagon-like peptide-1 receptor agonists (GLP-1RAs) have emerged as promising treatments for HO in CP patients. The mechanism involves increased pre-ingestive satiation via GLP-1 receptor neurons of the dorsomedial hypothalamus (DMH) [57]. GLP-1RAs activate metabolic and cardiovascular-related hypothalamic neurons, enhancing sympathetic activity [56]. Clinical evidence, although still limited, supports their potential across age groups. A case series of four females with CP-related HO treated with semaglutide for six months demonstrated significant weight loss (average 17% or 20.2 kg), with reductions in fat mass and improvement in energy levels and mobility [58]. The CRANIOEXE randomized controlled trial in adults demonstrated weight reductions with exenatide compared to placebo (−3.8 kg vs. −1.6 kg), although the adjusted treatment difference did not reach statistical significance (− 3.1 kg, 95% CI − 7.0 to 0.7, *p* = 0.11) [59]. A systematic review reported mean weight reduction of 7.4 kg across studies, with 85.7% of patients experiencing weight loss, in a pooled population spanning ages 13–71 years [60]. In younger populations (ages 10–26), exenatide extended-release significantly decreased energy intake during buffet meals (−430 kcal) compared to placebo [61]. Whether responses to GLP-1RAs differ by age remains uncertain: the available studies are small and heterogeneous, several cohorts described as supporting efficacy in younger patients included adults or participants with childhood-onset disease studied in adulthood [60, 61], and the adult CRANIOEXE trial did not demonstrate superiority over placebo for its primary weight outcome [59]. Direct age-stratified comparative trials are needed before differential efficacy can be concluded [5, 6]. The main adverse effects of GLP-1RAs include nausea and vomiting, with no major safety concerns reported [60]. In the CRANIOEXE trial, adverse events were more common with exenatide (95% of participants) versus placebo (70%) [59]. 

#### Insulin-sensitizing agents and central appetite regulators

Combined diazoxide–metformin therapy has shown efficacy in obese adolescents with CP, resulting in significantly reduced weight gain during treatment compared with the pre-treatment period (+ 1.2 ± 5.9 kg vs. + 9.5 ± 2.7 kg over 6 months, *p* = 0.004) [62]. The melanocortin-4 receptor agonist setmelanotide demonstrated promising results in a phase 2, open-label trial of mostly pediatric and young adult patients (ages 6–24 years) with acquired hypothalamic obesity, and a randomized phase 3 trial including adults is ongoing; adult-specific data therefore remain limited [63, 72]. 

#### Novel and investigational therapies

Emerging unimolecular polypharmacology approaches demonstrated enhanced efficacy. Tirzepatide, a dual GLP-1/GIP receptor agonist has been used successfully off-label for HO [64]. Other innovations include GLP-1/glucagon dual agonists and GLP-1/GIP/GCG triple agonists, which may offer a superior weight loss compared to GLP-1 monotherapy [65]. Orforglipron, a daily oral GLP-1 receptor agonist, represents a non-injectable alternative under investigation [66]. These approaches offer hope for managing this challenging condition in CP patients, with ongoing research needed to optimize age-specific treatment protocols and long-term outcomes.

### Bariatric and metabolic surgical interventions

Bariatric surgery is considered for CP patients with severe obesity resistant to conventional treatments [7, 29]. Current indications include BMI ≥ 95th percentile, crossing > 2 BMI percentiles on growth charts, and/or hyperphagia symptoms [67]. The 2022 American Society for Metabolic and Bariatric Surgery (ASMBS) and International Federation for the Surgery of Obesity and Metabolic Disorders (IFSO) updated guidelines have expanded the indications beyond those established 30 years ago [68]. However, careful evaluation is necessary given the unique challenges of hypothalamic damage in these patients. Growth completion is considered an important factor in pediatric patients, with some experts recommending bariatric intervention primarily after growth termination in cases of morbid obesity with severe complications [69]. 

Multiple surgical approaches have been employed in CP-related HO patients, options include Roux-en-Y gastric bypass (RYGB), sleeve gastrectomy (SG), laparoscopic adjustable gastric banding (LAGB), and biliopancreatic diversion (BPD) [70, 71]. Less invasive alternatives such as duodenal-jejunal bypass sleeve (DJBS, EndoBarrier) represent minimally invasive, reversible options that may be suitable for younger patients [70]. The selection criteria remain individualized based on patient characteristics, severity of obesity, and comorbidities.

Pediatric bariatric surgery requires specialized multidisciplinary expertise and dedicated adolescent programs, as emphasized in current guidelines [74]. For adolescents, surgery has been performed in the 13–18 year age range [73]. The 2018 ASMBS pediatric guidelines provide specific recommendations for this population [74], and many experts argue for completion of growth plate closure prior to intervention [69]. 

Bariatric surgery produces significant weight loss in CP-related HO patients, though typically less pronounced than in patients with common obesity. At 5-year follow-up, mean weight loss of 22.0% has been reported in CP patients versus 29.5% in matched controls [71]. Weight loss outcomes vary by procedure, with similar results between CP patients and controls for sleeve gastrectomy (21.7% vs. 21.8%), but less favorable outcomes with RYGB (22.7% vs. 32.0%) [71]. Psychosocial benefits include improvements in self-worth [73]. Reported complications include rare absorptive problems but generally stable endocrinological hormone replacement [71]. **Mechanistic case observations further show preserved postprandial GLP-1 secretion after bariatric surgery in CP-related obesity**,** supporting a physiological basis for its efficacy** [85].

## Cardiometabolic outcomes and long-term consequences

### Cardiovascular risks and events

Hypertension is a frequent comorbidity, occurring together with abdominal obesity and dyslipidemia as components of the metabolic syndrome, which affects 40–52% of long-term survivors (Table 4) [81]. Adults with CP face a 2.2-fold higher risk of early death compared to the general population [75]. Cardiovascular risk is increased by a history of stroke and hyperlipidemia, while cerebrovascular risk is elevated by higher BMI and radiotherapy exposure [75]. 

**Table 4:** Cardiometabolic Outcomes and Quality of Life

Outcome Domain	Pediatric Population	Adult Population
Cardiovascular Risk	Limited specific data on cardiovascular events; higher BMI z-scores correlate with hypothalamic damage severity [77]	2.2-fold higher risk of early death compared to general population; increased risk with hyperlipidemia and radiotherapy exposure. [75]
Metabolic Dysfunction	NAFLD affects 47% of survivors, with 27% developing hepatic steatosis within one year post-surgery [47]	Obesity prevalence increases from 37% to 53% post-treatment (*p* = 0.008); [44] MAFLD in 67.4% of adult-onset patients, with 32.5% at risk of advanced fibrosis [50]
Glucose Metabolism	Insulin resistance in 30% of cases; hyperinsulinemia contributes to weight gain [78, 104]	9.3-fold higher risk of diabetes mellitus compared to general population [75]
Dyslipidemia	Growth hormone discontinuation predicts decreased HDL-C levels [77]	Occurs in ~ 39% of patients in mixed suprasellar cohorts; characterized by reduced HDL-C and hypertriglyceridemia [78, 82]
Quality of Life	Significantly impaired with hypothalamic involvement; obesity associated with fragmented sleep, daytime sleepiness, and fatigue [87]	Physical limitations, especially in those with BMI > 30; employment and social functioning affected [107]
Educational/Vocational	Only 44% achieve tertiary education vs. 51% of other cancer survivors; 14% require special education services [107]	Employment challenges related to physical limitations and executive dysfunction [107]
Survival Impact	20-year overall survival lower with hypothalamic involvement (84% vs. 95% without involvement) [1]	Limited specific data on survival differences by hypothalamic involvement

CP treatment, particularly involving surgery and radiotherapy, has been linked to both short-term and long-term vascular complications. Surgical resection poses an immediate risk of intraoperative vascular injury, given the tumor’s close proximity to the circle of Willis and major cerebral vessels, which can result in acute ischemic stroke or hemorrhage. In contrast, radiotherapy is associated with delayed-onset vasculopathy, including arterial stenosis, moyamoya-like changes, and an increased risk of cerebrovascular events such as ischemic stroke, often manifesting years after treatment. These risks are particularly pronounced in pediatric survivors, necessitating long-term cerebrovascular surveillance [8]. Hypothalamic damage related to treatment correlates with morbid obesity and aggravates the lifelong risk of vascular events [8]. Beyond vascular injury, echocardiographic follow-up studies also demonstrate cardiac remodeling in childhood-onset survivors, underscoring the breadth of cardiovascular risk in this population [84]. Postoperative hypothalamic damage directly predicts weight gain in adult-onset CP patients [76]. In pediatric patients, the extent of hypothalamic involvement is prominently associated with higher BMI z-scores, highlighting the anatomical basis of treatment-related obesity [77]. 

### Metabolic dysfunction

Adults with CP exhibit a 9.3-fold higher risk of diabetes mellitus compared to the general population, with increased waist circumference further elevating the risk [75]. Pediatric patients demonstrate insulin resistance in 30% of cases, often linked to rapid postoperative weight gain [78]. Hyperinsulinemia arising from hypothalamic damage contributes to leptin resistance and reduced energy expenditure in both cohorts, though adults show more pronounced β-cell dysfunction [79, 80]. 

Nonalcoholic fatty liver disease (NAFLD) affects 47% of pediatric craniopharyngioma survivors, with 27% developing hepatic steatosis within one year post-surgery [47]. In adults, hepatic involvement is increasingly recognized: metabolic dysfunction-associated fatty liver disease (MAFLD) affected 67.4% of adult-onset patients in a recent cross-sectional study, with 32.5% of those affected at risk of advanced fibrosis [50]. Metabolic syndrome appears in 40–52% of long-term survivors and correlates strongly with levels of abdominal obesity and dyslipidemia [81, 82]. Pediatric NAFLD manifests earlier than population averages: in Jung et al.‘s cohort of 75 patients operated on before age 15 years, 68.4% of the 19 patients with complete anthropometric follow-up showed a postoperative BMI z-score increase (mean Δz = 1.13; range, 0.10–2.84), a magnitude of weight gain well beyond typical pediatric obesity trajectories and one that likely precipitates early hepatic steatosis in this population [47]. 

Dyslipidemia occurs in approximately 39% of patients in mixed pediatric suprasellar-tumor cohorts, characterized by reduced HDL-C and hypertriglyceridemia [78, 82]. Growth hormone (GH) deficiency is among the most common endocrine sequelae of CP and independently exacerbates the adverse metabolic phenotype, including dyslipidemia, visceral adiposity, and reduced lean mass [43, 75]. GH replacement improves body composition and lipid profiles and may have a protective effect on HDL-C levels, whereas a longer period off GH replacement after discontinuation during the transition period independently predicts declining HDL-C [77]. The metabolic effects of GH replacement in HO are nonetheless complex: because GH antagonizes insulin action, treatment may transiently reduce insulin sensitivity, a consideration of particular relevance in patients who are already insulin resistant, warranting glycemic monitoring during dose titration [29, 43]. Age-specific considerations are most pressing during the pediatric-to-adult transition (15–25 years), when GH replacement is frequently interrupted and deficiencies of GH and sex steroids may compound weight gain and cardiometabolic risk; structured transition care with retesting for adult GH deficiency and continuation of replacement when confirmed is therefore advocated [43, 77]. In adults with CP receiving GH replacement, registry data associate excess morbidity primarily with conventional cardiovascular risk factors and hypothalamic damage rather than with GH replacement itself [75]. Dietary interventions show limited efficacy, with only 31% of pediatric patients benefiting from caloric restriction versus 69% from carbohydrate modification [2]. Pharmacological approaches (GLP-1 agonists, metformin) yield modest results, necessitating multimodal strategies [6, 79]. 

For pediatric patients, particularly those with hypothalamic obesity following CP resection, annual monitoring of BMI z-scores, liver enzymes, and lipid profiles is essential to detect and manage early metabolic disturbances, including NAFLD and dyslipidemia. This is especially important during growth hormone (GH) therapy transitions, as shown in long-term follow-up studies [47, 77]. In adult patients with CP, regular monitoring of waist circumference and HbA1c is essential due to the high risk of progressive central obesity and emerging glucose intolerance. Although specific intervals are not universally defined, quarterly assessments are a pragmatic approach during the postoperative and early follow-up period, particularly when adjusting hormonal therapies. Clinical studies report a mean postoperative weight gain of ~ 4%, which often precedes the delayed onset of diabetes mellitus, typically manifesting months or years after surgery. These findings underscore the need for proactive and individualized metabolic surveillance in adult survivors [3, 44]. Both populations benefit from dual-energy X-ray absorptiometry (DXA) scans to assess and monitor visceral obesity and transient elastography for NAFLD staging [83, 86]. The convergence of hypothalamic damage, hormonal deficiencies, and obesity creates distinct metabolic trajectories, mandating age-tailored surveillance to mitigate long-term morbidity [79, 80]. 

### Psychosocial outcomes and quality of life

CP patients with hypothalamic involvement experience significantly impaired quality of life characterized by obesity, physical fatigue, reduced motivation, dyspnea, diarrhea, and non-optimal psychosocial development [1]. In pediatric patients, HO is frequently accompanied by fragmented sleep, excessive daytime sleepiness, and fatigue, creating a complex interplay of symptoms that negatively impact overall functioning [87]. This relationship between hypothalamic tumor involvement and these impairments suggests potential direct neurological mechanisms underlying psychosocial difficulties [87]. 

Several craniopharyngioma-specific studies have demonstrated persistent psychosocial and quality-of-life impairments in pediatric survivors: Sands et al. (2005) administered the SF-36 v2 or Child Health Questionnaire alongside the Achenbach Child Behavior Checklist to 29 survivors (12 F, 17 M; mean age at diagnosis 8 ± 3.8 y) after a mean follow-up of 6.8 ± 3.5 y, reporting low-average overall physical QOL with average psychosocial QOL and behavioral difficulties, including internalizing, attention, somatic, and social problems, with retrochiasmatic tumor location, recurrence, and additional surgery linked to poorer outcomes, whereas hydrocephalus, tumor size, and sex were not prognostic, and both physical and psychosocial QOL improved with longer postoperative intervals [88]. Poretti et al. (2004) followed 25 children (mean diagnosis age 9 y 2 mo, SD 4 y 3 mo) for a mean of 11 y 3 mo post-radical excision using semi-structured interviews and standardized questionnaires (PedsQL, CBCL, Epworth Sleepiness Scale), finding persistent school performance problems in 50% and marked deficits in social and emotional functioning domains leading to substantially reduced health-related QOL compared to normative values [89]. Pierre-Kahn et al. (2005) prospectively studied 14 children undergoing intended “total” resection, of whom 12 (85.7%) developed a postoperative hypothalamic syndrome characterized by hyperphagia and obesity, and despite normal intellectual function at 2-year follow-up, 80% exhibited impaired social integration, difficulty forming peer relationships and participating in group activities, and academic failure necessitating special education [90]. And Zada et al. (2013) conducted a systematic review of 11 studies encompassing up to 222 survivors per outcome, reporting overall neurobehavioral dysfunction in 57% (51/90), social impairment in 41% (91/222), school dysfunction in 35% (48/136), and emotional/affective dysfunction in 40% (58/146), with health-related QOL affected in 52% (49/95) and common issues including irritability, impulsivity, aggressiveness, and emotional outbursts, underscoring the need for routine neurobehavioral and psychosocial screening in pediatric CP survivors [91]. 

## Future directions and research needs

### Novel therapeutic approaches

Intranasal oxytocin, which modulates hypothalamic satiety circuits, demonstrated mixed efficacy in clinical trials for management of HO (Table 5). A 2023 randomized crossover trial in HO patients (median age 15.3 years) showed no significant weight difference versus placebo (− 0.6 kg, 95% CI: −2.7–1.5) [92]. A 2018 case report documented 13.5% BMI reduction over 38 weeks when combining oxytocin with naltrexone in a pediatric CP patient [93]. This suggests potential synergy between oxytocinergic and opioidergic pathways that merits further study, particularly in younger populations with acquired hypothalamic damage.

**Table 5:** Emerging Pharmacologic Therapies Targeting Hypothalamic Obesity

Therapeutic Class	Example Agents	Mechanism of Action	HO-Relevant Target	Stage of Development
Ghrelin Pathway Modulators [108, 109]	LEAP-2, (D-Lys3)-GHRP-6	Ghrelin receptor antagonism (GHS-R1a inverse agonism)	Appetite suppression, reduced hyperphagia	Preclinical, early translational
β3-Adrenergic Receptor Agonists [110]	Mirabegron	Brown adipose tissue activation and thermogenesis	Corrects low energy expenditure in HO	Approved for OAB; studied for obesity
FGF21-Based Dual Agonists [108, 111, 112]	ZT003 (GLP-1/FGF21 dual agonist)	Enhances insulin sensitivity, reduces steatosis and body weight	Targets central + peripheral metabolism	Preclinical/early clinical
Advanced Amylin Therapies [113, 114]	Pramlintide, Amylin-leptin combinations	Restores leptin sensitivity; VMH-targeted neurodevelopment in pediatrics	Synergistic satiety signaling	Clinical trials in general obesity
PPAR Pathway Modulators [115]	Tesaglitazar, Liposomal PPARα/γ agents	Anti-inflammatory + insulin sensitizing effect on adipose macrophages	Addresses inflammation and insulin resistance	Investigational
Central Insulin Sensitizers [116]	Phytohormone-based (e.g., Compound X)	Increases hypothalamic insulin receptor and downstream PI3K/mTOR signaling	Targets central insulin resistance	Experimental (animal studies)
Oxytocin–Naltrexone Therapy [93]	Oxytocin + Naltrexone	Modulates satiety and reward pathways; synergistic anorexigenic effect	Shown effective in pediatric HO case	Case report; early-phase exploration
Combination Therapies [94]	Amylin-leptin, GLP-1/GIP/GCG triple agonists	Multimodal targeting of satiety, thermogenesis, metabolism	Superior efficacy in treatment-resistant HO	Phase 1–2 trials
Tesofensine (Tesomet) [117–119]	Tesofensine + Metoprolol	Inhibits presynaptic reuptake of dopamine/serotonin/noradrenaline; reduces caloric intake	Monoaminergic pathways compensating for low sympathetic activity	Phase 2 HO trial (24 wk); Phase 3 planned
Melanocortin-4 Receptor Agonists [72]	Setmelanotide	Potent MC4R agonism restores melanocortin signaling to suppress appetite and increase energy expenditure	MC4R deficiency in injured hypothalamic and extra-hypothalamic circuits	Phase 2 open-label; international Phase 3 ongoing
Phentermine–Topiramate Combination [120, 121]	Phentermine, Topiramate	Sympathomimetic appetite suppression (phentermine) plus GABAergic modulation (topiramate)	Low sympathetic tone; hyperphagia and hypersomnolence	Approved for obesity; not yet formally studied in HO

Amylin-leptin combination therapy presents another innovative approach. Preclinical models revealed amylin pretreatment restores leptin sensitivity in diet-induced obese rats through enhanced STAT3 signaling in ventromedial hypothalamic nuclei [94]. A 24-week clinical trial demonstrated 12.7% weight loss with pramlintide/leptin coadministration versus 8.4% with either monotherapy [94], though this remains untested specifically in hypothalamic obesity. Diazoxide choline extended-release (DCCR) reduced hyperphagia severity by 28% in Prader-Willi syndrome patients over 52 weeks but has not yet been trialed in HO, although its mechanism aligns with the pathophysiology of hypothalamic obesity [95]. 

These findings underscore the complexity of targeting hypothalamic circuits in obesity management and highlight the need for novel, mechanism-driven therapies. While current pharmacologic strategies show promise in isolated cases or general obesity populations, few have been systematically evaluated in hypothalamic obesity cohorts. However, recent advances in translational neuroscience and endocrine pharmacology have led to a wave of experimental compounds that directly modulate hypothalamic satiety, thermogenesis, inflammation, and reward pathways. The following table outlines key therapeutic classes currently under investigation, each with potential applicability to the unique pathophysiology of HO.

### Gaps in knowledge and research priorities

Significant research gaps exist in understanding hypothalamic obesity across age groups in craniopharyngioma patients. Prospective comparative studies between pediatric and adult populations are notably lacking [8, 96], limiting insight into age-specific disease mechanisms and treatment responses. Research is hampered by inconsistent outcome measures across studies [2, 97]. For example, pediatric studies typically report BMI standard deviation (z-) scores whereas adult studies report absolute BMI or percentage weight change (e.g., ≥ 5% gain) [1, 3], and definitions of HO itself range from radiological grading of hypothalamic involvement to purely clinical obesity thresholds, precluding pooled analyses [10, 29]. Standardization beyond BMI to include metabolic parameters, eating behaviors, and hypothalamic function would enable meaningful cross-study comparisons and better assessment of interventions [98]. 

Current literature predominantly features short-term outcomes, highlighting the need for long-term follow-up data [71]. Understanding the longitudinal safety and efficacy of treatments, particularly newer agents like GLP-1 receptor agonists and Setmelanotide, remains limited [96, 98]. Comprehensive registry development, similar to the CAHtalog model [99], would facilitate valuable longitudinal data collection. Increased detail of surgical and radiosurgical treatment modalities will also offer greater insight into reduction of HO (Tables 6 and 7).

**Table 6:** Evolving Surgical and Radiotherapeutic Approaches to Preserve Hypothalamic Integrity in Craniopharyngioma

Approach Category	Technique/Technology	Key Features & Benefits
Posterior Hypothalamus-Sparing [122]	Hypothalamus-sparing resection (KRANIOPHARYNGEOM 2007)	Preserves posterior nuclei → higher QoL; significantly lower BMI z-scores at one year (1.89 SD vs. 2.92 SD after extensive resection). This approach reduces the incidence of severe obesity (28% vs. 54%) and increases the likelihood of maintaining normal BMI (38% vs. 17%) [102]
Limited Resection + Focused RT [123]	Subtotal resection + adjuvant radiotherapy	Maintains oncologic control with reduced hypothalamic injury
Endoscopic Endonasal Surgery (EES) [124, 125]	Endoscopic approach via natural nasal corridor	Direct sellar/suprasellar access; GTR ~ 64.1% with acceptable complication rates
Intraoperative Ultrasound (IOUS) [126, 127]	Real-time ultrasound during EES	Differentiates tumor subtypes, guides safe resection margins
“One-and-a-Half” Transcavernous Approach [128]	Interdural pituitary transposition and rotation	Preserves stalk & portal system; high GTR (Gross Total Resection) rates; avoids diabetes insipidus and hypopituitarism
Pituitary Transposition [125]	Intradural/extradural stalk mobilization	Aims to optimize tumor exposure while preserving endocrine function
Proton Beam Therapy (PBT) [129]	Particle RT for residual disease	Reduces off-target dose; comparable 5-yr EFS (92% vs. 91%) with potential for fewer late toxicities
Gamma Knife Radiosurgery (GKRS) [130]	Stereotactic RT for residual/recurrent tumor	5-yr PFS 70%, 10-yr PFS 43.8%; OS 91.5% & 83.9% at 5 and 10 years, respectively
BRAF/MEK Inhibitor Neoadjuvant Therapy [131, 132]	Targeted therapy for BRAFV600E-mutant papillary CP	Up to 90% tumor shrinkage at 5 months; 94% partial response rate; can obviate extensive surgery
Fluorescence-Guided Surgery [133, 134]	Tumor-specific fluorescent probes	Enhanced intraoperative visualization → potentially better preservation of hypothalamic tissue
Neuronavigation & Real-Time Imaging [135]	Advanced navigation + intraoperative imaging	Improves spatial orientation and surgical precision, minimizing unnecessary manipulation of hypothalamus

**Table 7:** Anatomical Classification Systems for Craniopharyngioma and Their Implications for Hypothalamic Obesity (HO)

Classification System	Categories	Key Features	HO Risk Implication
QST Classification	Type Q (pituitary region), Type S (pituitary stalk), Type T (tuberal/3rd ventricle floor)	Type T shows pia mater disruption and finger-like projections into 3rd ventricle	Type T carries highest hypothalamic injury and HO risk
Endoscopic (Stalk-based)	Peripheral (71.7%) vs. Central tumors	Peripheral subtypes more amenable to stalk preservation	Central tumors have lowest stalk preservation rate (15.4%) and highest HO risk
Growth Pattern (Pan et al.)	Group I (infrasellar), Group II (suprasellar extraventricular), Group III (suprasellar subpial/ventricular)	Group III involves both children and adults	Group III: 41.7% of children and 16.7% of adults show significant postoperative weight gain
Hypothalamic Location	Type A (infra-hypothalamic), Type B (perforating), Type C (supra-hypothalamic)	Informs surgical route selection (endonasal for Types A/B; transcranial for Type C)	Long-term control: 100% Type A, 92% Type B, 83% Type C; papillary CP highest in Type C

Despite HO’s substantial impact on daily functioning, quality of life and patient-reported outcomes remain underrepresented in research frameworks [2, 100]. Incorporating these measures would provide critical insights into the lived experience of patients and inform more holistic treatment approaches.

### Limitations

This review has several limitations. First, as a scoping review, it was designed to map the breadth of the available evidence rather than to appraise study quality; no formal risk-of-bias assessment was performed, and, because the literature was searched iteratively, no formal count of screened records is reported. Second, the underlying literature is highly heterogeneous: studies differ in populations, definitions of hypothalamic involvement and of HO itself, outcome measures (BMI, BMI z-scores, or percentage weight change), treatment protocols, and follow-up duration, which precludes quantitative synthesis. Third, no prospective cohorts directly comparing childhood-onset and adult-onset patients exist; all pediatric-versus-adult comparisons presented here are indirect, derived from separate studies, and should be regarded as hypothesis-generating. Fourth, several emerging therapies are supported only by case reports, small open-label series, or trials conducted in non-CP populations, and publication bias favoring positive findings cannot be excluded. Finally, this is a rapidly evolving field, and ongoing trials (for example, the phase 3 setmelanotide program) may modify the conclusions presented here.

## Conclusion

HO following craniopharyngioma demonstrates age-dependent differences in prevalence, pathophysiology, and clinical course. Pediatric survivors experience earlier and more severe weight gain, whereas adult-onset cases exhibit milder obesity trajectories but substantial cardiometabolic morbidity. Although small case series and mechanistic data suggest that responses to pharmacotherapies such as GLP-1 receptor agonists may differ by age at onset, direct comparative evidence is limited, the available cohorts are small and heterogeneous, and randomized trials have not yet demonstrated statistically significant weight reduction in either age group; conclusions regarding differential treatment response should therefore be regarded as preliminary. Mechanistic insights highlighting disrupted melanocortin signaling, central insulin/leptin resistance, and neuroinflammation nonetheless inform rational treatment strategies, and integrating early intervention, personalized pharmacotherapy, and surgical options may mitigate long-term cardiometabolic complications and improve quality of life in both patient populations. Future research should prioritize prospective, age-stratified comparative cohorts with standardized outcome measures; long-term safety and efficacy data for emerging pharmacotherapies; and the identification and validation of biomarkers of hypothalamic injury and treatment response, such as imaging-based grading of hypothalamic damage and circulating inflammatory markers, to guide individualized management.

## Data Availability

No datasets were generated or analysed during the current study.

## Declarations

### Generative artificial intelligence use

Generative artificial intelligence was used to assist with language editing, formatting, and citation consistency checking during the preparation of this manuscript. All scientific content and citations were reviewed and verified by the authors, who take full responsibility for the integrity of the work.
